# Supplementary material for: Morphological and Transcriptome Analysis of Wheat Seedlings Response to Low Nitrogen Stress
Source: Plants (Basel). 2019 Apr 15;8(4):98. doi: 10.3390/plants8040098 (PMC6524375; doi:10.3390/plants8040098)
Supplement: Supplementary file 1 [file plants-08-00098-s001.zip › plants - 461072 supp/Supp for proof.doc]

Supplementary material

Morphological and Transcriptome Analysis of Wheat Seedlings Response to Low Nitrogen Stress

**Table S3.** GO enrichment analysis for DEGs in wheat seedlings under low nitrogen stress.

| Categories | Description | GO | DEGs | | Genome | | Significant | |
| --- | --- | --- | --- | --- | --- | --- | --- | --- |
|  |  |  | Count | Percent  (%) | Count | Percent  (%) | *P* value | -Log_10_  (*P* value) |
| Biological process | alpha-amino acid metabolic process | GO:1901605 | 35 | 2.1 | 571 | 0.9 | 0.0012 | 2.9281 |
|  | carboxylic acid metabolic process | GO:0019752 | 79 | 4.8 | 1919 | 2.9 | 0.0100 | 1.9983 |
|  | glycine metabolic process | GO:0006544 | 10 | 0.6 | 26 | 0.0 | 0.0000 | 6.5436 |
|  | metabolic process | GO:0008152 | 92 | 56.1 | 30895 | 46.7 | 0.0000 | 11.1002 |
|  | organic acid catabolic process | GO:0016054 | 23 | 1.4 | 188 | 0.3 | 0.0000 | 6.4437 |
|  | organic acid metabolic process | GO:0006082 | 82 | 5.0 | 1951 | 2.9 | 0.0032 | 2.4921 |
|  | oxidation-reduction process | GO:0055114 | 293 | 17.8 | 5967 | 9.0 | 0.0000 | 26.2480 |
|  | response to abiotic stimulus | GO:0009628 | 66 | 4.0 | 962 | 1.5 | 0.0000 | 9.6635 |
|  | single-organism metabolic process | GO:0044710 | 422 | 25.7 | 11382 | 17.2 | 0.0000 | 14.9101 |
|  | small molecule metabolic process | GO:0044281 | 132 | 8.0 | 3171 | 4.8 | 0.0000 | 5.2233 |
| Molecular function | nitrite reductase activity | GO:0098809 | 3 | 0.2 | 4 | 0.0 | 0.0228 | 1.6421 |
|  | oxidoreductase activity | GO:0016491 | 244 | 14.9 | 5360 | 8.1 | 0.0000 | 17.1068 |
|  | dioxygenase activity | GO:0051213 | 18 | 1.1 | 130 | 0.2 | 0.0000 | 5.7825 |
|  | carboxy-lyase activity | GO:0016831 | 31 | 1.9 | 227 | 0.3 | 0.0000 | 11.1373 |
|  | glycerate kinase activity | GO:0008887 | 3 | 0.2 | 3 | 0.0 | 0.0058 | 2.2366 |
|  | lyase activity | GO:0016829 | 61 | 3.7 | 940 | 1.4 | 0.0000 | 8.1337 |
|  | vitamin transporter activity | GO:0051183 | 3 | 0.2 | 3 | 0.0 | 0.0058 | 2.2366 |
|  | aldehyde-lyase activity | GO:0016832 | 6 | 0.4 | 30 | 0.0 | 0.0314 | 1.5025 |
|  | dioxygenase activity | GO:0051213 | 18 | 1.1 | 130 | 0.2 | 0.0000 | 5.7825 |
|  | calcium ion binding | GO:0005509 | 57 | 3.5 | 1059 | 1.6 | 0.0000 | 4.5834 |
| Cellular component | cell part | GO:0044464 | 600 | 36.5 | 16952 | 25.6 | 0.0000 | 20.2984 |
|  | membrane | GO:0016020 | 609 | 37.1 | 21062 | 31.8 | 0.0004 | 3.3768 |
|  | cell | GO:0005623 | 615 | 37.4 | 17271 | 26.1 | 0.0000 | 21.7399 |
|  | organelle | GO:0043226 | 483 | 29.4 | 13190 | 19.9 | 0.0000 | 17.7496 |
|  | protein complex | GO:0043234 | 149 | 9.1 | 2900 | 4.4 | 0.0000 | 13.9788 |
|  | granum | GO:0009542 | 4 | 0.2 | 4 | 0.0 | 0.0001 | 4.2725 |
|  | stromule | GO:0010319 | 8 | 0.5 | 17 | 0.0 | 0.0000 | 6.3990 |

(*p-*value < 0.05) (leaf).

**Table S4.** GO enrichment analysis for DEGs in wheat seedlings under low nitrogen stress.

| **Categories** | **Description** | **GO** | **DEGs** | | **Genome** | | **Significant** | |
| --- | --- | --- | --- | --- | --- | --- | --- | --- |
|  |  |  | **Count** | **Percent**  **(%)** | **Count** | **Percent**  **(%)** | ***P* value** | **-Log_10_**  **(*P* value)** |
| Biological process | alpha-amino acid metabolic process | GO:1901605 | 60 | 3.6 | 571 | 0.9 | 0.0000 | 16.7235 |
|  | carbohydrate metabolic process | GO:0005975 | 93 | 5.6 | 2897 | 4.4 | 0.0300 | 1.5236 |
|  | carboxylic acid metabolic process | GO:0019752 | 94 | 5.6 | 1919 | 2.9 | 0.0000 | 5.9788 |
|  | cellular metabolic process | GO:0044237 | 638 | 38.2 | 20946 | 31.7 | 0.0000 | 5.3969 |
|  | metabolic process | GO:0008152 | 906 | 54.3 | 30895 | 46.7 | 0.0000 | 6.8447 |
|  | organic acid catabolic process | GO:0016054 | 39 | 2.3 | 188 | 0.3 | 0.0000 | 20.6216 |
|  | organic acid metabolic process | GO:0006082 | 96 | 5.8 | 1951 | 2.9 | 0.0000 | 6.2541 |
|  | oxidation-reduction process | GO:0055114 | 216 | 12.9 | 5967 | 9.0 | 0.0000 | 4.3969 |
|  | single-organism metabolic process | GO:0044710 | 371 | 22.2 | 11382 | 17.2 | 0.0000 | 4.3325 |
|  | response to organonitrogen compound | GO:0010243 | 9 | 0.5 | 37 | 0.1 | 0.0002 | 3.6383 |
| Molecular function | carbon-nitrogen lyase activity | GO:0016840 | 33 | 2.0 | 133 | 0.2 | 0.0000 | 20.1612 |
|  | antioxidant activity | GO:0016209 | 53 | 3.2 | 766 | 1.2 | 0.0000 | 7.6498 |
|  | catalytic activity | GO:0003824 | 827 | 49.6 | 28359 | 42.9 | 0.0000 | 5.3655 |
|  | binding | GO:0005488 | 920 | 55.2 | 32413 | 49.0 | 0.0001 | 4.2076 |
|  | protein kinase activity | GO:0004672 | 177 | 10.6 | 4954 | 7.5 | 0.0006 | 3.2007 |
|  | peroxidase activity | GO:0004601 | 53 | 3.2 | 714 | 1.1 | 0.0000 | 8.7799 |
|  | dioxygenase activity | GO:0051213 | 16 | 1.0 | 130 | 0.2 | 0.0001 | 4.1811 |
|  | calcium ion binding | GO:0005509 | 77 | 4.6 | 1059 | 1.6 | 0.0000 | 13.1035 |
|  | phosphotransferase activity | GO:0016773 | 184 | 11.0 | 5343 | 8.1 | 0.0035 | 2.4510 |
| Cellular component | extracellular region | GO:0005576 | 82 | 4.9 | 1937 | 2.9 | 0.0005 | 3.3098 |

(*p-*value < 0.05) (root).

**Table S5. Reference genome information.**

| Genome | Triticum_aestivum.TGACv1.dna.toplevel.fa |
| --- | --- |
| Genebuild by | Ensembl |
| Database version | 38 |
| Base Pairs | 13,427,942,385 |

**Table S6.** Primer information for qRT-PCR.

| **Primer** | **Forward (5´→3´)** | **Reverse (5´→3´)** |
| --- | --- | --- |
| Q-PsbP | GGTGACAGGGCAGTACATCGTCG | GGCATTTCATGTGAACTTGAACG |
| Q-MYB | CAAGGAGGAGGACGACCTCA | GCTGAGATGCGAGTTCCAGT |
| Q-Glycine | TGATGTCCTAGCCATACACGG | GCAGTTGTTGGCCTTCCAAA |
| Q-Catalase | AGACCCACATCCAGGAGAAC | AGCGTGTAGGTGTTGACGC |
| Q-WRKY | TTATTGCCTCCCCCTTCACC | TCCCAAGAGATGGTGGAACTG |
| Q-Asparagine | ACGCCAATGTTCCTGATGTC | GGTGCCTTGTGGAAGTAGAG |
| Q-Peroxidase | CCTCCGTCTCTTCTTCCACG | GACGTTCGGTTCCACATCCT |
| Q-MYB33 | ATATCGGCTCCTCGGCTAGT | CTGGATGAACACCGCTACGA |
| Q-RLI | TTGAGCAACTCATGGACCAG | GCTTTCCAAGGCACAAACAT |

CK

N-

CK

N-

**Figure S1.** View of wheat seedlings growth in CK and N- groups.
